# Modulating the Configurations of “Gel-Type” Soft Silicone Rubber for Electro-Mechanical Energy Generation Behavior in Wearable Electronics

**DOI:** 10.3390/gels9090686

**Published:** 2023-08-25

**Authors:** Vineet Kumar, Md. Najib Alam, Manesh A. Yewale, Sang-Shin Park

**Affiliations:** School of Mechanical Engineering, Yeungnam University, 280 Daehak-Ro, Gyeongbuk, Gyeongsan 38541, Republic of Korea; vineetfri@gmail.com (V.K.); mdnajib.alam3@gmail.com (M.N.A.); maneshphd@gmail.com (M.A.Y.)

**Keywords:** wearable electronics, “gel-type” soft silicone rubber, diatomaceous earth, multi-wall carbon nanotube

## Abstract

Electro-mechanical configurations can be piezo-electric transducers, triboelectric generators, electromagnetic induction, or hybrid systems. Our present study aims at developing energy generation through the piezoelectric principle. Gel-type soft SR with Shore A hardness below 30 was used as a versatile material for an elastomeric substrate. Also, multi-wall carbon nanotube (MWCNT), and diatomaceous earth (DE) were used as reinforcing fillers. This “gel-type” soft SR has crosslinking polymer networks with silicone encapsulated within its structure. Mechanical properties such as modulus or stretchability are of utmost importance for such devices based on “gel-type” soft. From the experiments, some of the mechanical aspect’s values are summarized. For example, the stretchability was 99% (control) and changes to 127% (3 phr, MWCNT), 76% (20 phr DE), and 103% (20 phr hybrid). From electro-mechanical tests, the output voltage was 0.21 mV (control) and changed to 0.26 mV (3 phr, MWCNT), 0.19 mV (20 phr DE), and 0.29 mV (20 phr hybrid). Moreover, from real-time biomechanical human motion tests in “gel-type” soft-based composites, a relationship among output voltage from machine to human motions was established. Overall, these configurations make them promising against traditional portable devices such as batteries for small power applications such as mobile phones.

## 1. Introduction

The “gel-type” soft SR-based wearable electronics are a hot topic of research both in industry and academics. This is due to their promising applications such as flexible devices and mechanical stretchability [1,2]. Some commercial devices falling under wearable electronics include smart watches [3] or electronic sensors for health monitoring such as heartbeat [4] or e-skin [5], etc. In addition, the emergence of artificial intelligence such as the Internet of Things [6] or machine-to-human interfaces is a hot topic of research recently [7]. Wearable electronic devices are smart in nature and can be worn on the body to monitor intelligent activities such as body joint movements [8]. These devices are user-friendly and equipped with sensors, software, and wireless connectivity to monitor human activities [9]. The popularity of these wearable devices is due to their multi-functionality [10], which is evident in various forms such as smartwatches and health monitors [3,4,5,11]. 

These wearable electronic devices are fabricated from stretchable substrates such as elastomers like silicone rubber (SR) [12]. The SR generally has “gel-type” soft properties, and these features make it useful for a wide range of applications such as wearable electronics. These multi-functional features of “gel-type” soft SR are due to its shore A hardness below 30, lightweight, flexible, twistable, stretchable, and tuning of properties according to needs. For example, the reinforcing filler particles can be added to tune its hardness making it from “gel-type” soft to stiffer for high-load applications. These dielectric SR matrices are electro-active and frequently used as substrates in these devices [13]. Moreover, the conductive fillers are added in SR to make them electrically conductive and make them useful for making the substrate electrically conductive. Most often, the conductive fillers are nano-carbon black [14], carbon nanotubes [15], or graphene [16]. These wearable electronic devices recently attracted material scientists and engineers globally due to their ability to mimic electro-mechanical aspects from machine to bio-mechanical from human motions [17]. These devices are lighter in weight and highly sensitive to the human body’s external mechanical or thermal stimuli or bio-mechanical motions [18].

The aspects of energy harvesting in these devices are generally related to principles based on piezoelectricity [19]. The mechanism involves the relation of developed dipoles originating in the dielectric substrate and results in an electric polarization against continuous external mechanical load [20]. Thus, these devices are a hot research topic due to their easy fabrication, easy process, and renewability [21]. In addition to these advantages, there are a few limitations related to the device’s stable output voltage or durability [22]. Therefore, new methodologies and updated innovative technology are required to harness energy from these devices by overcoming the disadvantages [23]. Moreover, the performance of such devices is correlated with the physical properties of materials used in the fabrication of such devices. So, versatile materials should be used in order to harness energy with an order of magnitude and superior durability [24].

The silicone rubber is divided into two categories depending upon the type of vulcanization. These are room temperature vulcanized (RTV) SR or one-component SR system, and high temperature vulcanized (HTV) SR or two-component SR system [25]. In the present work, RTV-SR was used which is because of its easy processing, high durability, and aging resistance [26]. Moreover, the SR has “gel-type” soft properties and is used in soft applications such as wearable electronics or tissue engineering [26]. Some key characteristics of “gel-type” soft properties are: (a) the durometer use shows that the SR rubber is soft with a Shore A hardness below 30; (b) the RTV-SR used in this work is generally transparent, soft, flexible, bendable, twistable, and stretchable; (c) SR is both viscous and elastic in nature such as liquid gel-like in the non-vulcanized state, semisolid state when mixed with MWCNT or DE and soft-solid after vulcanization with hardness below 30. Moreover, it recovers its original state after external stress is removed; (d) gel-type SR exhibit good damping properties such as it can absorb or dissipate mechanical vibrations, shocks or impacts; (e) gel-type SR has high thermal stability, and it can withstand high and low-temperature properties without noticeable damage; (f) gel-type SR are electrically insulating with high dielectric strength and low electrical conductivity. This property makes it suitable for wearable electronics, or insulation for sensitive components. 

In addition to the “gel-type” soft SR, the type of filler is of much importance. In the present work, MWCNT and DE and their hybrids are used to reinforce rubber, making them useful for these smart devices. Among them, MWCNT is frequently used as conductive material due to its high aspect ratio, and it is easily dispersible in the elastomeric substrate [27]. Moreover, the DE is also of importance due to its ability to reinforce the SR matrix as demonstrated in the present work. Additional benefitting reasons for using DE are its vast occurrence and its cost-effectiveness [28]. In this study, a versatile pathway for harnessing mechanical motions into electrical energy based on the piezoelectric principle was reported. The mechanical properties such as stretchability and stiffness were investigated from a potential wearable electronics perspective. Then, electro-mechanical experiments were performed to harness the electric energy at a compressive strain from 10–30%. The output voltage obtained from the universal testing machine was correlated with the voltage obtained from bio-mechanical motions obtained from body parts. These aspects of mimicking machine-to-human motion, especially for fillers like DE have not been understood fully and thus explored in this work. Moreover, the MWCNT–DE hybrid synergistically emerges as a promising candidate for obtaining higher output voltage, optimum stiffness, good stretchability, improved tensile strength, and efficient durability. To sum up, the output experimental results obtained from this work can be useful for commercial use, for example, the practice in self-powdered wearable electronic devices and health monitoring aspects or e-skin. Overall, the “gel-type” soft SR offers unique and balanced properties with robust flexibility, and softness with Shore hardness below 30, making it promising for soft applications like tissue engineering, or wearable electronics practiced in this work.

## 2. Results and Discussion 

### 2.1. Schematic Details of Different Aspects of the Work

**Figure 1:**
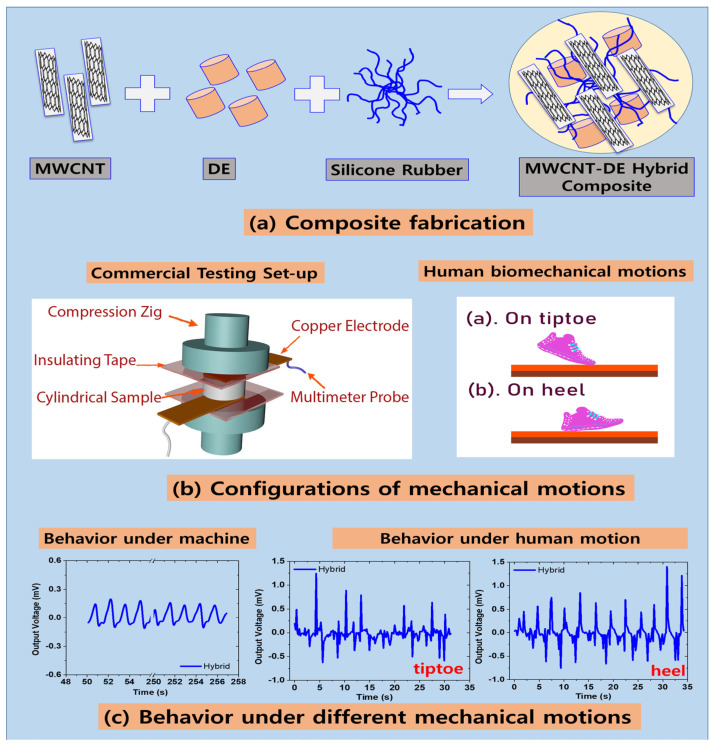
Different aspects of the present work.

Figure 1a shows the steps for the fabrication of the composites based on “gel-type” soft SR matrix and DE, MWCNT as fillers. In a hybrid composite, the MWCNT and DE were mixed together with the “gel-type” soft SR matrix. After cross-linking and molding, the samples were tested for properties and energy-harvesting applications. The brief procedure for making composites based on a “gel-type” soft SR matrix can be referred to the Section 4.2. in the experimental section. Figure 1b provides the configurations of the machine set-up to harness the energy based on the “gel-type” SR matrix through UTS machine or human motions such as tiptoeing and heels. Finally, the mimicking of the UTS machine output voltage with human motions was provided in Figure 1c. The hybrid filler content of 1+19 phr (MWCNT+DE) with a total of 20 phr was used in composites shown in Figure 1c for both machine and human motion experiments. The behavior of voltage generation from machines agreed with those obtained from human motions. The details of these aspects were provided in the result and discussion sections below.

### 2.2. Mechanical Properties of Composites

**Figure 2:**
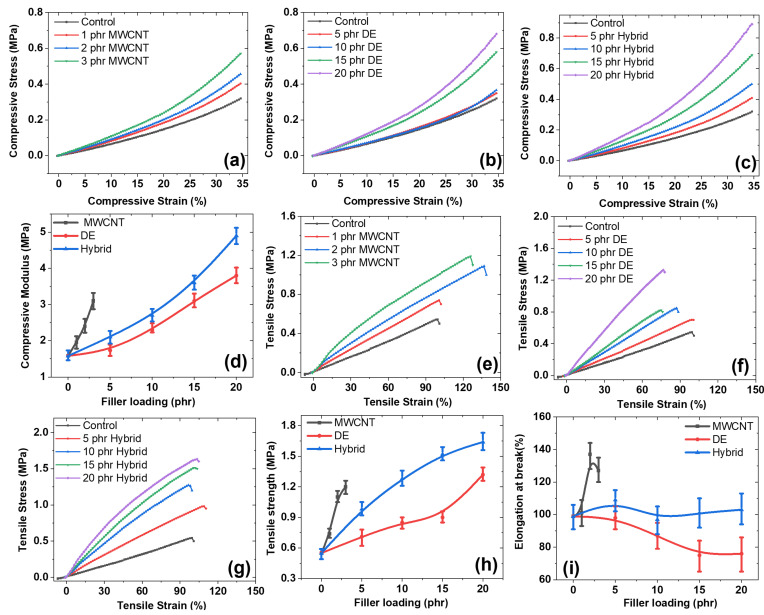
(**a**–**c**) Stress–strain of different filled composites under compressive mode; (**d**) modulus of different fillers in composites; (**e**–**g**) stress–strain under tensile mode for different fillers; (**h**) tensile strength of filled composites based on “gel-type” soft SR; (**i**) fracture strain of filled composites based on “gel-type” soft SR.

The representative mechanical properties such as stress–strain for composites based on “gel-type” soft SR matrix were studied under a maximum compressive strain of 35% and presented in Figure 2a–c. The mechanical performance shows that the reinforcing properties increase as the strain magnitude increases reaching a maximum of 35% strain. Such behavior could be accounted to be based on the reinforcing effect of the filler inside rubber composites [29,30]. Another reason could be due to the higher packing fraction of the microstructures inside the composites with increasing compressive strain [31]. In addition, the hybrid-filled composites based on “gel-type” soft SR show promising reinforcing properties that are higher than MWCNT and DE as the only filler. This could also be hypothesized because of the higher synergistic effect of the hybrid fillers in the composite [32]. It can also be witnessed that there is a sudden increase in stress values in DE-filled composites after 10 phr loading. This could be due to the establishment of percolative filler networks [33] in DE-filled composite after 10 phr loading. Overall, the soft “gel-type” behavior helps the SR matrix helps in maintaining the low stiffness of the composites, making them suitable for various soft applications such as tissue engineering or flexible electronics.

Figure 2d shows the features of modulus from different composites fabricated in this work. The results show that replacing 1 phr of DE with MWCNT was a novel choice that not only improves the modulus but also improves other properties like the fracture strain of composites. The soft SR matrix leads to lower stiffness as shown for the control sample, which is promising for “gel-type” soft applications. However, this stiffness is not useful for heavy load applications. For that, the reinforcing fillers are added to improve modulus and thus overall properties. So, this work presents the combination of mechanically soft “gel-type” composites like in the control sample of stiff composites like MWCNT and DE-filled samples. Overall, the hybrid composite emerges as a promising candidate in reinforcing the matrix among all fillers studied. For example, the modulus for different samples was 1.58 MPa (control) and increase to 3.1 MPa (MWCNT at 3 phr), 3.8 MPa (DE at 20 phr), and the highest for hybrid filled composite with 4.9 MPa at 20 phr. Moreover, it is witnessed from experimental data that the modulus improves with higher filler content in composites based on “gel-type” soft SR. This behavior is due to the physical and chemical interactions of fillers with a rubber matrix [34]. The quantity and quality of these interactions improve with an increase in filler loading [35]. Moreover, the lower modulus for the control sample based on “gel-type” soft SR is due to the absence of filler particles in the composite and thus mechanical properties were lower.

The representative stress–strain curves under tensile mode were tested and presented in Figure 2e–g. As witnessed above, the control sample consists of gel-type” soft SR and it is useful for soft applications while reinforced SR with higher mechanical stiffness is useful for heavy-load applications. From experimental examinations, it was witnessed that the mechanical properties depend mainly on the type and concentration of filler used as a reinforcing agent in the composite. For example, the MWCNT act as a good reinforcing filler while DE needed a higher concentration to improve the reinforcing properties. However, the hybrid filler using MWCNT–DE not only improve the reinforcing properties, but it makes the composite based on “gel-type” soft SR and balanced with higher modulus, high tensile strength, and better fracture properties than DE as the only filler. For example, tensile strength was 0.55 MPa (control) and increased to 1.2 MPa (MWCNT at 3 phr), 1.32 MPa (DE at 20 phr), and the highest for a hybrid filled composite with 1.64 MPa at 20 phr. Moreover, in Figure 2f, the curves show that the reinforcing performance improves drastically after 10 phr content of DE in the composite based on “gel-type” soft SR. This could be because of forming percolative filler networks [36]. In addition to this, the addition of increasing concertation of the filler improves the reinforcing properties of the composites. This type of behavior can be due to improved interactions which improve with the addition of filler [37]. There is a certain concentration up to which the properties improve and beyond that, there is a fall in properties. The concentration up to which the properties are highest is known due to the attainment of filler percolation [36] and the fall of properties after that is due to filler aggregation [38]. Moreover, there are various advantages of “gel-type” soft SR such as its softness helps to achieve robust flexibility, ease to fold, easy to fabricate, high stretchability, and biocompatibility for soft tissue engineering. However, the limitations of “gel-type” soft SR include (a) poor mechanical strength, which makes it unfit for high load bearing applications, (b) poor tear strength and tensile strength, (c) “gel-type” soft SR is expensive thereby limiting it for large scale commercialization, (d) the long time required for curing, e.g., “gel-type” soft SR takes 24 h to cure and thus make it time-consuming and difficult to process for large scale commercial applications. For such aspects of “gel-type” soft SR, a hybrid filler system can be used to exhibit optimum properties desired for both soft and mechanically stiff composites.

Most interestingly, the behavior of tensile strength derived from the above stress–strain curves is presented in Figure 2h. The behavior was inconsistent with that of compressive modulus and fracture strain of the studied composites based on “gel-type” soft SR. Some interesting facts show that the MWCNT-filled composites improve the tensile strength drastically compared to DE-filled composites. As stated above, it is also interesting to note that high filler content is required to obtain promising reinforcement for DE-filled composites. This could be because of the effect of particle size and other morphological effects [39]. For example, the DE has particles of micron size while MWCNT has a diameter of tubes in nanometer scale, a favorable cylindrical morphology that can be easily dispersed, and a high aspect ratio. [39,40] Moreover, the hybrid-filled composites based on “gel-type” soft SR show outstanding tensile strength better than both DE and MWCNT as the only filler. This is expected to be because of the synergy among MWCNT and DE particles in hybrid form. The association of MWCNT and DE microstructures in hybrid composites was studied in detail using SEM microscopy. The micrographs show the intercalation of tubes in the pores of DE filler. It can be further hypothesized that DE and MWCNT were in affinity with each other, thereby improving the reinforcing properties. To sum up, the advantages and limitations of composites based on “gel-type” soft SR, include that the properties can be tuned by manipulating processing, filler concentration, and type of additives. By evaluating these factors, one can decide which type of properties are desired in the motivation of the research scholar. For example, for soft applications, one should use low filler content or cure “gel-type” SR or higher filler content for heavy load applications.

Finally, the fracture strain of the composites for different filler contents was studied and presented in Figure 2i. From experimental measurements, it was found that the MWCNT-filled composites show superior stretching ability than other composites. For example, the fracture strain was 99% (control) and changed to 127% (MWCNT at 3 phr), 76% (DE at 20 phr), and 103% for hybrid composites based on “gel-type” soft SR at 20 phr. The higher stretching properties for MWCNT samples are due to its (a) higher surface area, which provides a higher interface between filler to rubber particles [41]; (b) higher aspect ratio that helps in improving filler inter-particles interactions in the composite [42]; (c) favorable tube-shaped morphology that allows it to efficiently distributed, thereby improving flexibility [43]. Furthermore, replacing 1 phr of DE with MWCNT in hybrid composites based on “gel-type” soft SR also shows some interesting results. For example, the fracture strain was higher for all samples in hybrid composites as compared to DE as only filler. So, the hybridization of fragile DE with MWCNT was useful in improving the mechanical properties of the composite such as its flexibility. Overall, the tensile strength and fracture strain of “gel-type” soft SR are poor and this makes its use limited. There are various ways to improve these mechanical properties. Among them, one frequently used method is adding filler reinforcement or stiff filler at a low concentration like an addition of 3 phr MWCNT in the present work. So, the addition of filler improves the “gel-type” soft SR and makes it applicable for properties with a higher fracture strain and superior tensile strength.

### 2.3. Filler Dispersion through SEM Microscopy

**Figure 3:**
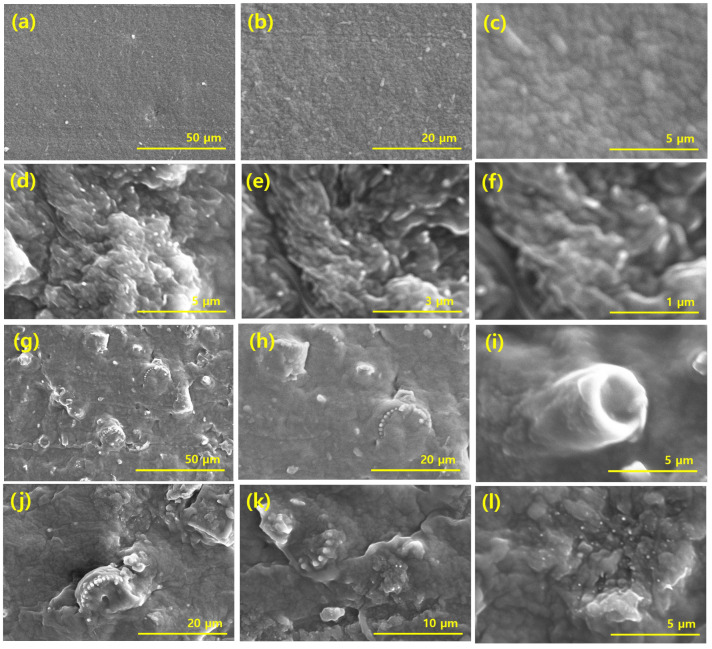
Filler dispersion through SEMs: (**a**–**c**) control sample with the “gel-type” soft SR; (**d**–**f**) MWCNT composite at 3 phr; (**g**–**i**) DE composites at 20 phr; (**j**–**l**) hybrid composites (MWCNT+DE with concentrations of 1+19 making it a total of 20 phr).

During composite preparation steps, the filler–rubber mixing and filler’s dispersion depend upon the type of method used during mixing. Moreover, the filler dispersion influences the final properties of the prepared composite [44,45]. So, it is necessary to study the dispersion of filler in rubber composite based on “gel-type” soft SR to support the behavior of their properties. Here, we employed SEM microscopy in order to investigate the filler dispersion at a microscopic scale. Figure 3a–c presents the SEM micrographs of the control sample of “gel-type” soft SR. All the images show the absence of filler particles in the composite as expected. Moreover, the higher resolution image (Figure 3c) shows the rough surface of the control sample. The images of the control sample also show a soft SR surface. The influence of “gel-type” soft SR depends on various factors such as the type of filler, content of filler, processing conditions, or type of vulcanization [26].

However, the “gel-type” soft SR has both positive and negative influences on filler dispersion in composites. Some positive effects are (a) “gel-type” soft SR provides good wetting and dispersion of the filler. This property can help to achieve better filler dispersion and hence better mechanical properties; (b) the use of “gel-type” soft SR helps in decreasing filler settling, especially for high-density filler such as iron particles thereby maintaining the properties and filler dispersion upon long-term storage. But the use of “gel-type” soft SR also has limitations such as (a) difficulty in mixing high filler loading, especially during solution mixing technique; (b) potential compatibility issues between “gel-type” soft SR and filler particles. It is necessary to functionalize the filler or rubber matrix to fix compatibility issues. Figure 3d–f shows the MWCNT dispersion in the SR matrix. Figure 3d justifies the uniform dispersion of MWCNT in “gel-type” soft SR. The SEM images further show that the MWCNT has good bonding with the rubber matrix. This supports the good reinforcing properties of MWCNT-filled composites. Also, the high-resolution images in Figure 3e,f show that the MWCNT tubes are protruding out of the matrix making the surface rough and effective. This behavior of MWCNT dispersion in SR agrees with the literature [46].

Furthermore, the DE dispersion in the SR matrix was presented by SEM images in Figure 3g–i. The appearance of DE particles in the SR matrix was lower compared to the number of MWCNT particles in Figure 3d–f. This difference could be due to the macroscopic size of DE particles, their three-dimensional nature, lower quantity, and porous morphology. The high-resolution images in Figure 3i show the adsorption of soft rubber particles on the surface of the DE particle. This feature supports the fragile reinforcing properties such as fracture strain of the DE-filed composites based on “gel-type” soft SR as shown in Figure 2i. The reason behind the adsorption of SR particles on the surface or DE could be because of the mechanically soft nature of the SR matrix with a hardness below 65 as supported by previous studies [47]. Finally, Figure 3j–l shows the dispersion of MWCNT and DE in hybrid form. The SEM micrographs show that the MWCNT particles are located in the vicinity of DE particles. In some cases, the MWCNT tubes are attached with a polymer shell around DE particles. This behavior is the reason behind the synergism among DE and MWCNT-filled hybrid composites based on “gel-type” soft SR as reported in mechanical properties and electro-mechanical properties. Moreover, it can be stated from SEM on hybrid fillers that introducing MWCNT in DE for hybrid composites was promising as DE particles help in improving the rubber–filler interface and their dispersion. Finally, “gel-type” soft SR is useful for making both soft and hard composites with Shore A hardness between 25–45. These composites are useful not only for soft applications like tissue engineering but also for high load-bearing applications. Especially, the addition of reinforcing fillers improves the hardness of the filled composites, and “gel-type” soft SR assists in achieving improved dispersion thereby leading to higher mechanical properties such as tensile strength and fracture strain. Moreover, the SEM images support the results obtained in Section 2.2 and Section 2.4 to Section 2.6 below.

### 2.4. Theoretical Models for Predicting the Mechanical Behavior of the Composites

**Figure 4:**
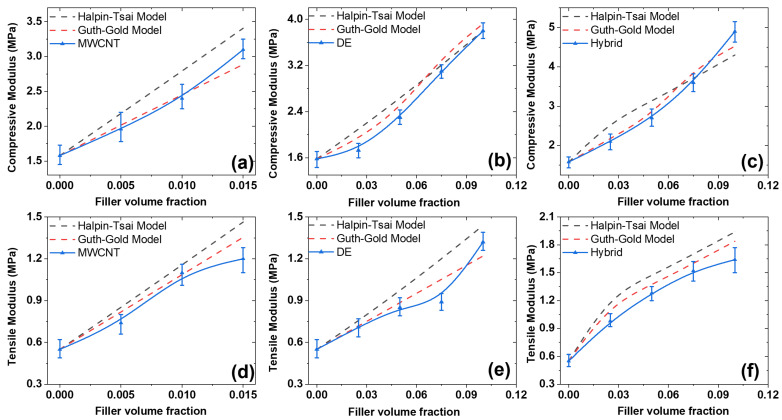
Experimental outcomes with theoretical models: (**a**–**c**) compressive modulus of different samples based on “gel-type” soft SR; (**d**–**f**) tensile modulus of the samples with different models.

The modulus of the filled composites strictly depends on the type of filler or the type of rubber matrix. Here, the “gel-type” soft SR was used in this work with hardness below 30 and it became stiffer with the addition of fillers such as DE or MWCNT. The validation of the experimental results with theoretical models is practiced and presented in Figure 4. This figure shows the compressive and tensile modulus is lower for the “gel-type” soft SR used in the control sample and it increases at higher filler content. This property of the control sample justifies that the control sample is soft while filled composites are stiffer. Most of these models consider the critical parameters of the experimental studies such as filler volume fraction, and the aspect ratio of the filler [48,49]. Fillers with two-dimensional (2-D) and 1-D morphology are often examined. Here, the 1-D MWCNT, 3-D DE, and their hybrids were used, and these filler particles were added to improve the reinforcing properties. The theoretical models such as Guth–Gold–Smallwood equations [50] or Halpin–Tsai equations [51] are well-known models in the literature that are used in predicting the modulus of the filled composites. They were traditionally developed for 0-dimensional materials such as carbon black but recently upgraded and become useful for 1-D, 2-D, and 3-D as well. Recently, Kumar et al. added interacting factor parameters for hybrid fillers, which are based on the rule of mixing fillers in rubber composites [46]. In hybrid composites based on “gel-type” soft SR, the interacting factor plays an important role in predicting the theoretical values from the equations. These interacting values are in the range of >0.1 and <0.9.

The following equations were used to predict the behavior of the modulus to validate the experimental results thus obtained. The Guth–Gold equations [46,50] were

E_MWCNT_ = E_o_ [(1 + 0.67 × f_MWCNT_ × ϕ_MWCNT_)] (1)

E_DE_ = E_o_ [(1 + 0.67 × f_DE_ × ϕ_DE_)] (2)

E_Hybrid_ = E_o_ [(1 + 0.67 × f_DE_ × ϕ_DE_) + (1 + 0.67 f_MWCNT_ × ϕ_MWCNT_)] × i (3)

Similarly, the equations employed in determining modulus using Halpin–Tsai equations [46,51] were

E_MWCNT_ = E_o_ [(1 + 2f_MWCNT_ × ϕ_MWCNT_)/(1-ϕ_MWCNT_)] (4)

E_DE_ = E_o_ [(1 + 2f_DE_ × ϕ_DE_)/(1-ϕ_DE_)] (5)

E_Hybrid_ = E_o_ [(1 + 2f_MWCNT_ × ϕ_MWCNT_)/(1-ϕ_MWCNT_) + (1 + 2 f_DE_ × ϕ_DE_)/(1-ϕ_DE_)] × i (6)

In Equations, (1)–(6), E_MWCNT_, E_DE_, and E_Hybrid_ were the theoretically predicted modulus for MWCNT, DE, and their hybrid. f_MWCNT_, f_DE_, and f_Hybrid_ were the theoretically assumed aspect ratio for MWCNT, DE, and their hybrid. Finally, ϕ_MWCNT_, ϕ_DE_, and ϕ_Hybrid_ were the filler volume fractions for MWCNT. DE, and their hybrid. The “i" is the interacting factor based on the rules of mixtures in hybrid composites [46]. The “E_o_” is the experimental value of unfilled rubber. It can be witnessed that the theoretical calculation is strictly influenced by the nature of the rubber matrix used in the composites. Here, the “gel-type” soft SR with lower hardness achieves the lower theoretical values as predicted and agrees well with the experimental values. From Figure 4a–f, it can be stated that in most cases, especially for MWCNT-filled composites, the predicted models agree very well under lower filler content but deviate largely at larger filler concentrations. This is because of different assumptions in the model and experimental conditions [52]. For example, both models assume perfect interfacial interactions and filler dispersion, but it is hard to achieve these assumptions experimentally. Moreover, after a certain higher concentration of filler, the filler percolation was achieved but this is not assumed in models. So, due to these reasons, the models deviate largely at higher filler content [53]. Finally, the Guth–Gold model was often closer to experimental values than those predicted with Halpin–Tsai equations.

### 2.5. Energy Harvesting of the Composites under Compressive Mode

**Figure 5:**
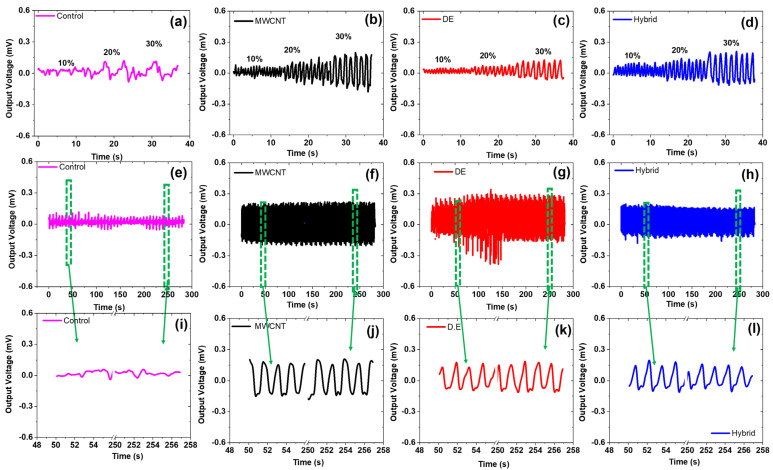
(**a**–**d**) The energy harvesting output for different composites under 10%, 20%, and 30% strain; (**e**–**h**) durability of the different composites at 30% strain; (**i**–**l**) insets for the initial and final durability cycles providing the behavior of the cycles, filler concentration was MWCNT composite at 3 phr, DE composites at 20 phr and hybrid composites (MWCNT+DE with concentrations of 1+19 making it a total of 20 phr).

The nature of “gel-type” soft SR can have a significant effect on electro-mechanical behavior and thus energy harvesting applications. Some points are (a) the soft nature of “gel-type” SR can help to achieve high flexibility, making it suitable for energy harvesting applications that involve mechanical movements under the piezo-electric principle; (b) the “gel-type” soft SR possesses good damping and isolation behavior, which is very useful in energy harvesting applications. For example, the “gel-type” soft SR can enhance the efficiency and reliability of the energy harvesting systems; (c) the “gel-type” soft SR can shield the sensitive electronic parts from moisture, dust, or other mechanical strains thereby improving the life span of the electronic device; (d) the “gel-type” soft SR has tunable properties which are soft at the control and become stiff upon addition of filler making them smart materials for energy harvesting applications. The electro-mechanical energy generation under mechanical strain or human motion is a hot topic of research recently and is thus explored experimentally in this work [54]. The sensitivity of the electro-active composite materials based on “gel-type” soft SR often exhibits piezo-electric behavior and often became a source of electricity [55]. Thus, these composites became self-powdered wearable electronic devices type for commercial applications such as sensitivity towards mechanical motions and useful for health monitoring such as heartbeat or e-skin [56,57]. Figure 5a–d shows the electro-mechanical features of the composites based on “gel-type” soft SR against increasing strain from 10–30%. Results show that the output voltage increases with increasing compressive strain from 10–30%. This behavior can be because of a decrease in inter-distances between filler particles and an increase in the density of filler networks of the composites [58]. This higher filler density such as an increase in the density of filler networks results in improved output voltage at the higher compressive strain of 30%. Another reason for a higher output voltage at a higher compressive strain is due to an increase in the electrical conductivity of the composites [58]. This feature is because of an increase in the packing of filler particles at a higher strain [59].

It is reported that electrical conductivity is directly correlated with output voltage [60] and thus output voltage is higher at 30% and lower at 10% strain. Moreover, it was also found that the hybrid filler composites based on “gel-type” soft SR show the highest output voltage at all compressive strains. The reason behind such an increase in output voltage for a hybrid filler system is due to synergism among the binary fillers. Finally, the poor output voltage for DE-based composites is due to their poor stretchability, fragile mechanical strength, large particle size, and poor tensile strength [61,62]. Among them, the large particle size and mechanical stretchability are critical to affect the electro-mechanical properties of the composites. For example, the large particle size leads to lower filler–polymer interfacial interactions that lead to small stress transfer when subjected to mechanical strain [63]. It is noteworthy that a device’s durability is important when discussing its commercialization [64]. So, the durability of different composites at 30% strain was shown in Figure 5e–h. The results show stable voltage output for different composites. Moreover, hybrid-filled composites based on “gel-type” soft SR show a higher and more stable voltage than other composites. It is known that the stability of output voltage is an issue for such energy-generating composite materials. Thus, hybrid-filled composites are advantageous in such disciplines. In addition, the higher voltage in hybrid composite was assumed because of the synergistic effect [65] among the DE and MWCNT as only fillers. It is also worth noticing that the “gel-type” soft SR-based control sample has specific effects on energy harvesting results. For example, the magnitude of the output voltage is influenced by the magnitude of compressive strain. This behavior was in agreements of filled composite samples. Moreover, the durability experiments show stable voltage output for the composite making it useful for the desired soft applications. Therefore, it is important to consider the addition of desired ingredients in the “gel-type” soft SR to obtain desired energy harvesting systems and output voltage.

**Scheme 1:**
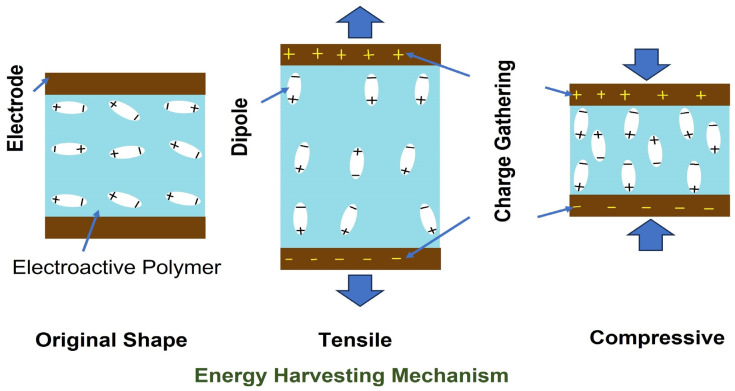
Piezo-electric mechanism of the composites.

**Figure 6:**
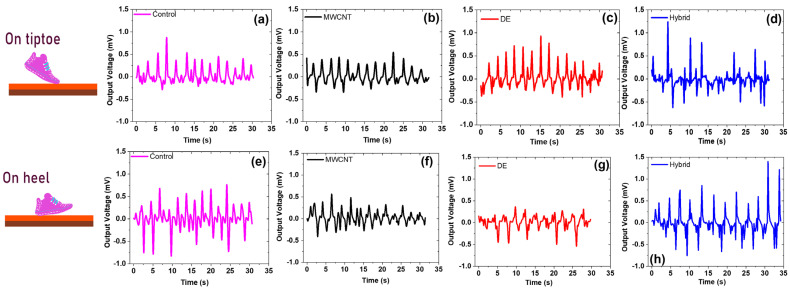
The biomechanical energy harvesting of different composites: (**a**–**d**) tiptoe pressing; (**e**–**h**) heel pressing; for both experiments, the MWCNT was 3 phr, DE was 20 phr, and hybrid was 1+19 phr (MWCNT+DE) that is a total of 20 phr.

Moreover, there is synergy in the distribution of MWCNT tubes in the vicinity of DE-based micron-size particles and intercalation of MWCNT tubes in pores of DE particles. It is interesting to state that the mechanical property, such as the tensile strength of hybrid filler, is higher than DE or MWCNT as the only filler. The higher tensile strength helps in overcoming the crack formation in the electrode or substrate of the device used in energy harvesting [66]. Thus, it is supporting higher energy generation with voltage stability. The pattern of the output voltage of the composites based on “gel-type” soft SR was further studied by zooming the initial and final cycles of the durability tests (Figure 5i–l). In almost all cases, it was noticed that the output voltage was stable, and the initial–final cycles were almost the same. It further justifies the usefulness of the composites prepared in the present work as promising energy-generating tools. Moreover, another reason for stable output is related to its low piezoelectric energy generation mechanism (Scheme 1). It is because piezoelectric materials are materials in which electricity is generated under mechanical deformation. Our composites mimic this piezoelectric principle and generate voltage under mechanical strain even without the addition of piezoelectric materials such as barium titanate or lead zirconate titanate. Thus, even the control sample shows output voltage in a small quantity. The higher voltage is generated in composites containing electrically conductive fillers such as MWCNT. The main mechanism behind these effects involves the displacement of electric diploes present in an electroactive silicone rubber matrix under mechanical deformation. Under further mechanical deformation, these dipoles are displaced in opposite directions causing a polarization effect within the substrate of the energy harvesting sample. With further mechanical deformation, the oppositely charged particles reach the conductive copper electrode causing a voltage generation. The magnitude of this voltage generation depends upon the nature of the substrate used in the energy harvesting sample. For example, the substrate containing electrically conductive fillers such as MWCNT generates higher voltage than the control sample. This can be demonstrated in Figure 5. Moreover, Figure 5a,e, and i show lower output voltages of the control sample than the other filled composites in Figure 5. Hence, the output voltage is stable and thus useful for self-powered wearable electronic devices. Overall, the “gel-type” soft SR offers many advantages for its use in energy harvesting devices for wearable electronics. These configurations are high flexibility, favorable damping properties, and other useful tunable properties, making them suitable for particular applications. However, considerations must be given to overcome limitations such as electro-mechanical stability for long life, optimum mechanical strength, and potential compatibility for making “gel-type” soft SR useful as energy harvesting systems. The relationship between the electromechanical behavior attained through the UTS machine was mimicked with that from biomechanical behavior through human motion in Figure 6 in Section 2.6.

### 2.6. Modulating the Configurations of the Machine with Human Motion

Energy generation through human motions in a compressive or tensile mode is a recent hot topic area of research [67]. These aspects are promising for self-powered wearable and flexible electronics. The “gel-type” soft SR can have a great positive effect when used in energy harvesting through human motion. Most efficiently, its property to tune its stiffness, robust stretchability, and light weight make it promising for wearable electronic material. Some other benefits for use in energy generation through human motion include: (a) the great comfort of “gel-type” soft SR makes it useful for energy generation; (b) the “gel-type” soft SR offers robust energy conversion efficiency making it a promising candidate for wearable electronics; (c) good durability, damping, and vibration absorption, and biocompatibility with low toxicity making it useful for tissue engineering applications. Here, the validation of the behavior of the output voltage results obtained from the UTS machine was performed through human motion. Figure 6a–d shows the biomechanical behavior of tiptoe pressing and Figure 6e–h for heel pressing. The experiments show that the biomechanical behavior of the composites based on “gel-type” soft SR agreed with those shown in Figure 5. For example, it was found that all the composites show a lower output voltage than hybrid-filled composites, which is the same as the output voltage of the machine. Also, the DE-filled composite shows poor output voltage than other composites.

The poor voltage of DE-filled composites based on “gel-type” soft SR is due to the poor tensile strength and fracture strength of this composite. These poor mechanical aspects lead to the development of cracks in the substrate or electrode of the device and thus lower output voltage [68]. It is also noteworthy that the output voltage is higher with heel pressing than with tiptoe pressing for all composites based on “gel-type” soft SR. It could be due to a higher magnitude of strain in heel pressing than those with tiptoe pressing. The higher magnitude of the strain in heel pressing is influenced by an improvement in electrical conductivity [69]. The higher electrical conductivity results in higher output voltage as both are correlated with each other. However, the voltage generation was less stable than those achieved with the machine, and further research is required in this aspect. Finally, the self-powered devices based on the above study were promising because of their versatile behavior and utmost sensitivity to external factors such as (a) the magnitude of strain, and (b) the type of material used in preparing the composite or (c) the area of the electrode. Overall, it is important to note that the desired properties of “gel-type” soft SR can be tuned by selecting the ingredients during the formulation and processing of the composites making them useful for both soft and stiff applications. However, proper optimization and engineering of the device based on “gel-type” soft SR need to be selected in capturing and utilizing human motion energy.

## 3. Conclusions

The “gel-type” soft SR offers a range of configurations that can be tuned from soft to stiff materials depending upon the area of interest. Its unique combination of properties includes high flexibility, robust stretchability, damping and vibration absorption, lightweight, and the ability to tune by optimizing ingredients. These aspects of “gel-type” soft SR make it promising and ideal for energy generation applications. The present work demonstrates the study of the possible relationship between electro-mechanical behavior from machine testing with biomechanical motions from human motions. These results were of great practical applications such as self-powered wearable electronics devices. The results of this work show that there was a further relation between mechanical properties with output voltage obtained from the devices based on the composites based on “gel-type” soft SR. For example, the tensile strength and fracture strain of hybrid composites were 1.64 MPa and 103%, respectively, and it attains higher output voltage for all samples studied. The higher tensile strength and fracture strain helps in healing the crack formation in the sample, which depletes the output voltage. The other mechanical properties were investigated. For example, the compressive modulus was 1.58 MPa (control) and increased to 3.1 MPa (3 phr, MWCNT), 3.8 MPa (20 phr DE), and 4.9 MPa (20 phr hybrid). Finally, it was found that the output voltage was higher for hybrid composite samples based on “gel-type” soft SR for both machine and human motion tests. Hence, the device based on composite samples studied in this work shows a great and versatile route for viable applications based on self-powered wearable electronics for health monitoring or e-skin. In future work, we will control both the magnitude of output voltage and its stability in human motion-type experiments for self-power wearable electronic devices. To sum up, “gel-type” soft SR holds great potential candidates for energy generation applications in wearable electronics. As far as further research on this subject is concerned, the research and development activities will continue to explore new ways to explore this hot topic with improved efficiency and reliable harvesting solutions for wearable electronics.

## 4. Materials and Methods

### 4.1. Materials

The silicone rubber with room temperature vulcanization (RTV-SR) feature was used as an elastomeric matrix in the present work. The commercial name of RTV-SR is “KE-441-KT”, and it was purchased from Shin-Etsu Chemical Corp. Ltd., Tokyo, Japan. The hardener with the commercial name “CAT-RM” was used as a curing agent and was purchased from Shin-Etsu Chemical Corp. Ltd., Japan. The MWCNT with the commercial name of “CM-100” was purchased from Hanwha Nanotech Corporation Ltd., Seoul, Korea. The used MWCNT has a diameter in the range of 12–15 nm, lateral dimensions from 500 nm to 1 µm, and a surface area of ~250 m^2^/g. The micron size diatomaceous earth was purchased from Sigma-Aldrich, St. Louis, MO, USA. The mold-releasing agent used to spray molds was obtained from Nabakem, Pyeongtaek-si, Korea.

### 4.2. Fabrication of Composites

The fabrication of the composites was optimized and summarized into 4 steps as described below.

Step-1: The different types of fillers were mixed with RTV-SR through solution mixing. Their concentration was reported in Table 1. The rubber–filler mixing lasted approximately 10 min at which a homogenous phase was achieved in the composite.
Step-2: Then, 2 phr of curing agent was added and the final composite was mixed again for 1 min before pouring into molds. These molds were then pressed mechanically and kept for 24 h at room temperature for curing.
Step-3: The samples were finally taken out from the molds and kept into deep freezing (< 0 °C) to inhibit over-curing. It was found that the composites are over-cured if kept at room temperature for more time because of the promotion of additional reactions in the presence of room temperature and moisture.
Step-4: Finally, the samples were taken out 24 h prior to testing for different reinforcing properties and applications. These applications were energy harvesting in compressive mode and mimicking the electromechanical behavior of samples and biomechanical behavior with tip-toe or heel.

**Table 1:** Fabrication of different composites.

Formulation	RTV-SR (phr)	MWCNT (phr)	DE (phr)	MWCNT+DE Hybrid (phr)	Vulcanizing Agent (phr)
Control	100	-	-		2
RTV-SR/MWCNT	100	1,2,3 * (0.005, 0.01, 0.015) **	-		2
RTV-SR/DE	100		5,10,15,20 * (0.027, 0.054, 0.081, 0.11) **		2
RTV-SR/Hybrid	100			1 + 4=5, 1 + 9 = 10, 1 + 14 = 15, 1 + 19 = 20 * (0.005+0.023, 0.005+0.05, 0.005+0.077, 0.005+0.1) **	2

* Filler loading in phr; ** filler loading in filler volume fraction.

### 4.3. Characterization Techniques of the Composites

The filler dispersion of composites was performed through SEM. The SEM microscope was S-4800, Hitachi, Tokyo, Japan. For preparing the sample, the cylindrical sample (thickness of 10 mm and diameter of 20 mm) was sliced into 0.2 mm thick and mounted on the SEM stub. Then, it was subjected to platinum coating for 2 min to make the surface conductive. The sample was finally subjected to SEM investigations for microscopic features of the composite. The mechanical properties of the composites were performed by employing a universal testing machine (UTS, Lloyd Instruments, Bognor Regis, UK) under a load cell of 1 kN for both compressive and tensile tests. The compressive mechanical properties were performed using above-mentioned same cylindrical sample. The compressive strain rate was 4 mm per minute, the pre-load of 0.5 N, and the strain of a maximum of 35% was applied. Then, the reinforcing properties such as stress–strain or compressive modulus were reported. The mechanical properties under tensile strain were performed on a dumbbell sample (gauge length of 20 mm, thickness of ~2 mm, width of 4 mm), a pre-load of 0.1 N, and strain rate of 200 mm per minute. DIN 53 504 standards were followed to measure these mechanical properties. The output voltage generation from these composites can be withdrawn using a multimeter with a commercial name of 34401A and purchased from Agilent Technologies Inc, Santa Clara, CA, USA.

## Data Availability

Data are available on reasonable request.
